# Molecular characterization of *Anaplasma platys *strains from dogs in Sicily, Italy

**DOI:** 10.1186/1746-6148-2-24

**Published:** 2006-07-26

**Authors:** José de la Fuente, Alessandra Torina, Victoria Naranjo, Silviane Nicosia, Angelina Alongi, Francesco La Mantia, Katherine M Kocan

**Affiliations:** 1 Department of Veterinary Pathobiology, College of Veterinary Medicine, 250 McElroy Hall, Oklahoma State University, Stillwater, OK 74078, USA; 2 Instituto de Investigación en Recursos Cinegéticos IREC (CSIC-UCLM-JCCM), Ronda de Toledo s/n, 13071 Ciudad Real, Spain; 3 Istituto Zooprofilattico Sperimentale della Sicilia, Via G. Marinuzzi n°3, 90129 Palermo, Italy

## Abstract

**Background:**

The genetic diversity of *Anaplasma platys *(Rickettsiales: Anaplasmataceae) strains is currently poorly defined. The present study was designed to characterize *A. patys *strains in dogs from Palermo, Sicily, Italy, using a combination of PCR and sequence analysis of the 16S rDNA, heat shock operon *groESL *and citrate synthase (*gltA*) genes.

**Results:**

Blood was collected from 344 dogs (111 pet dogs, 122 pound dogs and 111 hunting dogs) during 2003–2005 in the Province of Palermo, Sicily, Italy. The prevalence of *A. platys *in dogs in Sicily, as demonstrated by PCR and sequence analysis of the 16S rDNA, *groESL *and *gltA *genes, was 4%. None of the samples were positive for *A. marginale, A. centrale, A. ovis *and *A. phagocytophilum *DNA. Three different *gltA *genotypes of *A. platys *were identified in dogs from Sicily. Two of the *gltA *sequences of Sicilian *A. platys *strains were different from sequences reported previously. However, one of the *gltA*, 16S rDNA and *groESL *sequences were identical to the sequence of *A. platys *strains from other regions of the world characterized previously.

**Conclusion:**

At least three different strains of *A. platys *were identified in dogs from Sicily by PCR and sequence analyses of the 16S rDNA, *groESL *and *gltA *genes. The results reported herein suggested that genetic diversity of *A. platys *strains may be similar to *A. ovis*, but lower than the diversity reported for *A. marginale *and *A. phagocytophilum*. This lower genetic diversity may have resulted from restricted movement of infected hosts compared to *A. marginale*-infected cattle and/or the limited host range of *A. ovis *and *A. platys *as compared with *A. phagocytophilum*. These results expand our knowledge about *A. platys *and encourage further research for analysis of the genetic variation of *A. platys *strains worldwide.

## Background

The genus *Anaplasma *(Rickettsiales: Anaplasmataceae) contains obligate intracellular organisms found exclusively within membrane-bound inclusions or vacuoles in the cytoplasm of both vertebrate and invertebrate host cells [1,2]. This genus includes pathogens of ruminants, *A. marginale*, *A. centrale*, *A. bovis *(formerly *Ehrlichia bovis*), and *A. ovis*. Also included in this genus is *A. phagocytophilum *(previously recognized as *E. equi*, *E. phagocytophila *and the human granulocytic ehrlichiosis (HGE) agent), which infects a wide range of hosts including humans and wild and domesticated animals, and *A. platys *(formerly *E. platys*) which is infective for dogs.

In dogs, *A. platys *develops within platelets and is the etiologic agent of canine infectious cyclic thrombocytopenia, but infected dogs are usually asymptomatic [3]. Canine infections of *A. platys *have been reported throughout the world, including the United Sates [3-5], Spain [6,7], France [8], Greece [9], Italy [10], Taiwan [11], China [12], Thailand [13], Japan [14-16], Venezuela [13,17,18], and Australia [19,20]. However, *A. platys *infection is difficult to detect in vivo because the bacteremias are usually low [21-23]. Furthermore, serologic tests may be inaccurate because they are cross-reactive with other *Anaplasma *[8,21,24]. Recently, a PCR assay was optimized to allow for accurate identification of *A. platys *infection in dogs [20]. The PCR test, confirmed by sequence analysis of amplicons, is considered to be the most reliable diagnostic test for *A. platys *to date.

Despite the worldwide distribution of *A. platys*, limited information is available on the genetic diversity of *A. platys *strains [13,18,25]. Herein, we characterized strains of *A. platys *from dogs in Palermo, Sicily, Italy, using a combination of PCR and sequence analysis of 16S rDNA, heat shock operon *groESL *and the citrate synthase (*gltA*) genes.

## Methods

### Blood samples

Blood was collected from 344 dogs (111 pet dogs, 122 pound dogs and 111 hunting dogs) during 2003–2005 in the Province of Palermo, Sicily, Italy, for these studies. Blood was collected into sterile tubes with anticoagulant (EDTA), held at 4°C until arrival at the laboratory and then stored at -20°C for DNA extraction.

### DNA extraction, PCR and sequence analysis

DNA was extracted from blood and tick samples using the GenElute Mammalian Genomic DNA Miniprep Kit (Sigma, St. Louis, MO, USA). The *A. marginale/A. centrale/A. ovis *and *A. phagocytophilum msp4 *genes were amplified by PCR as reported previously [26,27]. The *Anaplasma *spp. 16S rDNA was amplified by PCR using oligonucleotide primers 16SANA-F (5'-CAG AGT TTG ATC CTG GCT CAG AAC G-3') and 16SANA-R (5'-GAG TTT GCC GGG ACT TCT TCT GTA-3') as described previously [28,29]. The *A. platys*-specific 16S rDNA, *groESL *and *gltA *PCRs were done as reported by Martin et al. [20] and Inokuma et al. [25], respectively. PCR reactions contained 2 μl (0.1–10 ng) DNA and 10 pmol of each primer in a 50-μl volume (1.5 mM MgSO_4_, 0.2 mM dNTP, 1X AMV/Tfl 5X reaction buffer, 5u *Tfl *DNA polymerase) employing the Access RT-PCR system (Promega, Madison, WI, USA). Reactions were performed in an automated DNA thermal cycler (Eppendorf Mastercycler^® ^personal, Westbury, NY, USA or Techne model TC-512, Cambridge, England, UK) for 35 cycles. Control reactions were done using the same procedures and reagents described above but without DNA added to the PCR reaction to rule out PCR contaminations. Carrying-over was ruled out due to the low number of PCR positive samples and the differences in the amplicon sequences. PCR products were electrophoresed on 1% agarose gels to check the size of amplified fragments by comparison to a DNA molecular weight marker (1 Kb Plus DNA Ladder, Promega).

Amplified 16S rDNA, *groESL *and *gltA *fragments were resin purified (Wizard, Promega) and cloned into pGEM-T vector (Promega) for sequencing both strands by double-stranded dye-termination cycle sequencing (Core Sequencing Facility, Department of Biochemistry and Molecular Biology, Noble Research Center, Oklahoma State University). At least two independent clones were sequenced. Multiple sequence alignment was performed using the program AlignX (Vector NTI Suite V 5.5, InforMax, North Bethesda, MD, USA) with an engine based on the Clustal W algorithm [30]. BLAST [31] was used to search the NCBI databases to identify previously reported sequences with identity to those obtained in the study described herein.

### Sequence accession numbers

The GenBank accession numbers for *gltA *sequences of *A. platys *strains are [GenBank: DQ525686–DQ525688].

## Results and discussion

### Prevalence of *A. platys *in dogs from Sicily

The observed prevalence of *Anaplasma *spp. was analyzed by PCR and sequence analysis of 16S rDNA amplicons. Of the 344 dogs analyzed, 14 (4%) were positive for *Anaplasma *spp. DNA (Table 1). Sequence analysis of 16S rDNA amplicons resulted in 100% identity to previously reported *A. platys *sequences. None of the samples were positive for *A. marginale, A. centrale, A. ovis *and *A. phagocytophilum *DNA.

Previous 16S rDNA PCR-based *A. platys *studies in dogs reported observed prevalences of 33% (9/27, North Carolina, USA [5]), 32% (64/200, Okinawa, Japan [15]), 45% (10/22, Central Australia [20]) and 16% (7/43, Lara, Venezuela [18]). The analysis reported herein included a greater number of samples but the observed prevalence of *A. platys *in dogs was lower than in previous studies. These results suggested that *A. platys *infection in dogs in Sicily may be very low. However, differences in the sensitivity of the PCR due to the size of the amplicon and primer sequences may affect the results of prevalence studies reported by different groups.

### Molecular characterization of *A. platys *strains from Sicily

The 14 positive dog samples were characterized with *A. platys*-specific 16S rDNA, *groESL *and *gltA *PCR and sequence analysis. All dogs had 16S rDNA and *groESL *sequences identical to [GenBank: AY530806] and [GenBank: AY848753] *A. platys *Spanish and Italian strain sequences reported previously, respectively. These results agree with previous reports in which little genetic diversity was observed between 16S rDNA and *groESL *sequences of *A. platys *strains [16,18,20].

The sequence of *A. platys gltA *resulted in 3 different genotypes (Table 2). The sequences of *A. platys *from samples Miky, Dog 4 and Dog 9 were present in 9/14, 3/14 and 2/14 of the positive dog samples, respectively. A single nucleotide change in Dog 9 sequence resulted in an amino acid change (Table 2). Although the information about *A. platys *sequences is limited, these results suggest that *gltA *sequences may be more diverse than 16S rDNA and *groESL *sequences.

The result of *gltA *sequence analysis suggested that at least three different genotypes of *A. platys *infect dogs in Sicily. Two of the *gltA *sequences of Sicilian *A. platys *strains (Dog 4 and Dog 9) were different from sequences reported previously (Table 2). However, the *gltA *sequence of Miky strain was identical to the sequence of the Sommieres French strain. Huang et al. [18] suggested that *A. platys *strains are not geographically segregated. Although limited by the number of sequences available, the results of our study suggested that *gltA *may provide some phylogeographic information about *A. platys *strains. Nevertheless, although 16S rDNA, *groESL *and *gltA *sequences may be useful for phylogenetic studies of *Anaplasma *spp. [25], they were not informative for phylogenetic studies of *A. platys *strains.

The genetic diversity of *A. marginale*, *A. phagocytophilum *and *A. ovis *strains have been documented using different genetic markers [27,29,32,33]. The results reported herein suggested that the genetic variation in *A. platys *may be similar to *A. ovis *but less than that observed in *A. marginale *and *A. phagocytophilum*. This low genetic variation may have resulted from restricted movement of infected hosts compared to *A. marginale*-infected cattle and/or the limited host range of *A. ovis *and *A. platys *as compared with *A. phagocytophilum *[33].

The tick vectors for the transmission of *A. platys *have not been extensively characterized. *Rhipicephalus sanguineus *[10,34], *Dermacentor auratus *[35] and *Hyalomma truncatum *[18] have been suggested as possible vectors of *A. platys*. However, experimental transmission of *A. platys *with these tick species has not been demonstrated [36]. Nevertheless, ticks of the genera *Rhipicephalus*, *Dermacentor *and *Hyalomma *are present in Sicily and could act as vectors of *A. platys *in this region [37].

## Conclusion

Low observed prevalence (4%) of *A. platys *was detected in dogs from Sicily by PCR and sequence analysis of 16S rDNA, *groESL *and *gltA *genes. Three different *gltA *genotypes of *A. platys *were identified in these dogs. The results reported herein suggested that genetic diversity of *A. platys *strains may be similar to *A. ovis *but lower than that for *A. marginale *and *A. phagocytophilum*. The lower genetic diversity of *A. platys *may have resulted from restricted movement of infected hosts as compared to *A. marginale*-infected cattle and/or the limited host range of *A. ovis *and *A. platys *as compared with *A. phagocytophilum*. These results expand our knowledge about *A. platys *and encourage further research to characterize genetic diversity of *A. platys *strains worldwide.

## Competing interests

The author(s) declare that they have no competing interests.

## Authors' contributions

José de la Fuente has designed and performed the sequence analyses and wrote the manuscript.

Alessandra Torina has organized and supervised the initial screening of dog samples by PCR.

Victoria Naranjo, Silviane Nicosia, Angelina Alongi and Francesco La Mantia have performed all the DNA extractions and PCR analyses.

Katherine M. Kocan has contributed to the final draft of the manuscript.

All authors have read and approved the final manuscript.

## Figures and Tables

**Table 1:** Characterization of dogs positive for *A. platys *DNA.

**Sample**	**Age (months)**	**Sex**	**Category**	**Sampling date**
Igor	60	Male	House dog	03.01.2004
432	60	Female	Pound dog	07.05.2004
8259	36	Female	Pound dog	07.05.2004
420	NR	NR	Pound dog	07.09.2004
467	96	Male	Pound dog	07.16.2004
517	NR	NR	Pound dog	07.03.2004
Miky	8	Male	House dog	08.30.2004
Nico	72	Male	House dog	08.26.2004
Barone 2	18	Male	Hunting dog	11.25.2004
Paco	12	Male	House dog	01.12.2005
Dog 4	12	Male	Pound dog	06.03.2005
Dog 7	12	Male	Pound dog	06.03.2005
Dog 8	12	Female	Pound dog	06.03.2005
Dog 9	1	Male	Pound dog	06.03.2005

Abbreviation: NR, not recorded.

**Table 2:** Nucleotide sequence differences among *gltA *from different strains of *A. platys*.

***A. platys *Strains**	**Nucleotide Positions**							
	
	330	421	483	518	921	1146	1212	1224
Spain [GenBank: AY530807]	G	A (T)	A	G (R)	T	T	G	T
Sommieres, France [GenBank: AB058782]	*	*	*	*	*	G	*	*
Okinawa, Japan [GenBank: AY077620]	*	*	*	T (L)	C	G	*	*
Miky, Sicily [GenBank: DQ525686]	*	*	*	*	*	G	*	*
Dog 4, Sicily [GenBank: DQ525687]	*	*	*	*	*	G	C	*
Dog 9, Sicily [GenBank: DQ525688]	A	C (P)	T	*	*	G	*	C

The numbers represent the nucleotide position starting at translation initiation codon adenine. Conserved nucleotide positions with respect to the Spanish strain are represented with asterisks. Amino acid changes are represented in parenthesis using the single letter code.
